# High -density lipoprotein cholesterol as a predictor for diabetes mellitus

**DOI:** 10.22088/cjim.9.2.144

**Published:** 2018

**Authors:** Hong Wu, Peng Ouyang, Wenjun Sun

**Affiliations:** 1 School of Medicine and Health Management, Tongji Medical College, Huazhong University of Science and Technology, Wuhan, China.; 2 School of Management, Harbin Institute of Technology, Harbin, China.

**Keywords:** Diabetes, Risk score, Score model

## Abstract

**Background:**

Diabetes is a prevalent chronic disease around the world. To evaluate the risk of diabetes comprehensively, we developed a score model for risk prediction with HDL-C as a protective factor.

**Methods:**

We extracted physical examination data of 2728 individuals. The data contain 18 demographic and clinical variables. To identify the statistical significant feature variables, the backward stepwise logistic regression was used based on the data of the “exploratory population”. To ascertain the cutoff value of the selected variables, we used the Youden index. Then we assigned each variable level a score according to the estimated regression model coefficients and then calculated the individual’s total score. We gained the cutoff value for the total score through the Youden Index and stratified the total score into four levels. We employed the data of “validation population” to test the performance of the score model based on the area under the ROC curve.

**Results:**

Age, LDL-C, HDL-C, BMI, family history of diabetes, diastolic blood pressure and TCHO were selected as statistically significant variables. The diabetes risk score range varied from 0 to 17. The risk level categorized by the total score was low, middle, high and extremely high, with a score range of 0-2, 3-7, 8-12 and 13-17, respectively.

**Conclusions:**

The score model based on physical examination data is an efficient and valuable tool to evaluate and monitor the potential diabetes risk for both healthy and unhealthy people at an individual level.

Diabetes mellitus is a prevalent chronic disease worldwide as a normal and serious health issue (1, 2). Studies showed that the prevalence of diabetes mellitus is becoming an urgent and important public health problem for Chinese adults (3). Diabetes can result in or promote the incidence of a set of complications, like depression (4, 5), diabetic retinopathy (6-8). Some studies have proven the association between intensive lifestyle intervention and the remission of type 2 diabetes (9). It has been proven that the prevention of the onset of type 1 diabetes or the reduction of the risk of type 2 diabetes through interventions were possible and feasible (10, 11). Now, the major concern for patients with diabetes, would be the individual diabetes risk evaluation and the related early implementation of health interventions. Physical examination is widely used to check up the personal physical condition. However, it is time-consuming and would lead to overload of work for the doctors since many of the medical examinations were performed at the end of month or year in China. Such a practical way of the self-health evaluation is of great importance to alleviate the medical resource strain and the doctor’s workload, especially for a poor and unevenly distributed medical resource environment in China. Many of the existing diabetes score models are based on the questionnaire or survey data (12, 13). 

Some were focused on the physiological parameter (14, 15). Research shows that the incidence of diagnosed type 2 diabetes for the people in Harbin, China has experienced a dramatical increase in recent years with the annual rate reaching 12% (16). The prevention of diabetes is of great importance and urgency. However, the diabetes risk pattern for the people in Harbin, northeastern China, which is a diabetes prevalent site, has not been studied.

The main goal for our research was to set up a comprehensive and ready-to-use scoring model to identify the risk factors of diabetes mellitus and construct a risk score according to the physical examination data. Also, we verified the scoring model performance with the data of a “validation population”.

## Methods

**Study design and population: **This was a methodological study which was designed for local doctors to help them evaluate the patient’s diabetes risk more easily and conveniently. We extracted the medical examination data of 2728 subjects with age greater than 20 in 2014 from the School Hospital in HIT. We assigned the subjects into two groups: the exploratory group and validation group. If the number of subjects distincted from the two groups, the robustness and performance of the score model would be affected heavily. To make our score model robust, we attempted to minimize the difference between the two goups when cutting them into two balanced parts. And to guarantee performance of the score model, the exploratory population was assigned some more subjects. In detail, among them, 1465 subjects were randomly selected into the “exploratory population”, based on which a score model was developed. The remaining subjects were used for the model validation as the “validation population”. The screening criteria of diabetes were focused on the fasting plasma glucose, with the level of fasting plasma glucose higher than 7.0mmol/L would be diagnosed as diabetes (17).

The research was approved by the Ethics Committee of the School Hospital of Harbin Institute of Technology. For confidetiality, all of the names and the medical exmination document numbers were deleted by the School Hospital of Harbin Institute of Technology.

**Statistical analysis** **:** The statistical analysis was performed with R program (18). All continuous data were expressed as the mean±standard deviation or median depending on normality. Differences between groups were assessed by the two-sample t-test. For categorical data, chi-square test was used for comparison. We initially selected 18 potential risk factors for the development of the score model. These potential risk factors were: age, gender, BMI, personal history of hypertension, personal history of coronary heart disease, personal history of cerebrovascular diseases, family history of hypertension, family history of diabetes, family history of coronary heart disease, family history of cerebrovascular diseases, smoking or not smoking, drinking or not drinking systolic blood pressure, diastolic blood pressure, triglyceride, Total Cholestrol (TCHO), High Density Lipoproteine Cholestrin (HDL-C), and Low Density Lipoproteine Chilostrin (LDL-C). A backward stepwise logistic regression model was used to screen out the statistically significant factors. A p-value of less than 0.05 was considered to be statistically significant. The significant factors were then used to construct the scoring model. Based on the receiver operation characteristic (ROC) curve of the selected variables, the cutoff value of each variable was obtained by calculating the Youden index to formulate the scale of the scoring model. We calculated the total score of each subject to better understand the risk of diabetes. The total score was then included into a binary logistic regression model and the Youden index was used to determine the cutoff value of the total score according to the ROC curve. Based on each subject’s total score, we divided the total risk into four status levels: low risk, middle risk, high risk and extremely-high risk. 

Score model test was important to check the accuracy or efficiency of the model. We validated the performance of the diabetes risk score model via the “validation population”. The area under the ROC curve (AUC) was usually used to test the accuracy of the score model. If AUC was larger than 0.5, it would be considered that the performance of the model is valid. First, we obtained the total score for each subject in the “validation population” based on the score model. We then calculated the area under the ROC curve (AUC) to evaluate the performance of the score model.

## Results

In both the exploratory and validation populations, most of the characteristics were non-significant except for family history of diabetes (table 1), which suggesting that the comparability between the two populations groups was rather good. By the logistic regression, the significant risk factors for the score model were age, LDL-C, BMI, family history of diabetes, HDL-C, diastolic blood pressure and TCHO (table 2). 

Among them, age, BMI, family history of diabetes, diastolic blood pressure and TCHO appeared to be risk factors because the related coefficients were positive while HDL-C and LDL-C appeared to be preventive factors due to their negative coefficients. Previous studies suggested that LDL-C was a risk factor for diabetes (19) while HDL-C was a preventive factor (20, 21), thus in our model, we considered the LDL-C as a risk factor. BMI was marginally significant and some studies showed that it was a significant risk factor for diabetes (22, 23), so we included it into our model. As shown in table 2, the AUC for the integrated model was 0.834 (95%CI, 0.802-0.867), which is much higher than the AUC of any single factor. It was suggested that we should evaluate the risk of diabetes by combining all the statistically significant, marginally significant factors together. 

**Table 1:** Basic demographic and clinical characteristics of the “exploratory population” and “validation population”.

	**Diabetes**			**Non-diabetes**		
**Characteristic**	**Exploratory** **group**	**Validation** **group**	**p-value**	**Exploratory** **group**	**Validation** **group**	**p-value**
n	105	105	--	1360	1158	--
Gender (male)	64	67	0.776	737	650	0.350
Age (years)	64.82±13.79	66.79±13.59	0.298	51.44±17.59	51. 46±17.62	0.978
BMI* (kg/m^2^)	26.31±2.90	26.35±3.16	0.921	24.35±3.52	24.35±3.42	0.958
Diastolic blood pressure (mmHg)	83.05±10.11	82.01±9.34	0.441	76.79±10.43	77.71±10.49	0.029
Family history of diabetes (Yes)	24	2	<0.0001	128	1153	<0.0001
LDL-C ** (mmol/L)	3.15±1.06	3.04±1.15	0.492	2.76±0.92	2.75±0.93	0.896
HDL-C *** (mmol/L)	1.57±0.42	1.60±0.49	0.639	1.83±0.54	1.81±0.51	0.349
TCHO **** (mmol/L)	5.17±1.19	5.11±1.41	0.739	4.86±0.92	4.83±0.93	0.504
FPG***** (mmol/L)	9.25±2.30	9.19±2.24	0.849	5.32±0.56	5.32±0.53	0.772

* Body Mass Index

** Low Density Lipoproteine Chilostrin

*** High Density Lipoproteine Cholestrin

**** Total Cholestrol

***** Fasting plasma glucose

**Table 2:** Backward stepwise logistic regression model and the cutoff values of related risk factors

**Variable**	**Coefficient**	**p value**	**Odds ratio**	**AUC(95% CI)**	**Cutoff value**
Age (years)	0.057	<0.0001	1.058	0.719(0.677-0.762)	53
LDL-C* (mmol/L)	-1.766	<0.0001	0.171	0.608(0.549-0.667)	2.98
BMI** (kg/m^2^)	0.071	0.0508	1.074	0.666(0.619-0.608)	23.6
Family history of diabetes (Yes)	1.235	<0.0001	3.437	0.567(0.526-0.608)	
HDL-C*** (mmol/L)	-2.643	<0.0001	0.071	0.645(0.541-0.705)	1.705
Diastolic blood pressure (mmHg)	0.033	0.0029	1.033	0.664(0.616-0.713)	75
TCHO**** (mmol/L)	1.817	<0.0001	6.152	0.579(0.517-0.641)	5.6
Area under the ROC curve	0.834, 95%CI (0.802-0.867)				

* Low Density Lipoproteine Chilostrin

** Body Mass Index

*** High Density Lipoproteine Cholestrin

**** Total Cholestrol

To better evaluate the effects of risk factors in the score model, we categorized the selected continuous factors, mainly age, BMI, LDL-C, HDL-C and diastolic blood pressure, into three levels according to the cutoff values as shown in table 2. For most of the selected factors, the higher the level was, the higher risk it presented, except for the preventive factor of HDL-C. For HDL-C, the level higher than the cutoff value of 1.705 was considered as the reference level. For TCHO, we categorized it into two levels due to the data restriction. The result of the categorization was shown in table 3. 

The score was attributed mainly from the β-coefficient. The principal of the score attribution was described as follows: β=0.01-0.2, the corresponding score was assigned 1; β=0.21-0.8, the score was 2; β=0.81-1.2, the score was 3; β=1.21-2.2, the score was 4; β>2.2, the score was assigned the highest of 5 (24). Based on these individual scores, we calculated the total score of the “exploratory population”, and obtained the cutoff value of the total score based on its ROC curve. 

The cutoff value of the total score was 7.5. We then categorized the total score into four levels for the risk stratification: low risk (the total score of 0-2), middle risk (3-7), high risk (8-12) and extremely-high risk (13-17).

**Table 3:** Logistic regression model with the stratified risk factors and the related scoring system.

	**Coefficient**	**Odds ratio (95% CI)**	**Score**
Intercept	-5.223		
Age (years)			
<=53	reference		0
54-68	1.116	3.051 (1.722-5.443)	3
69-91	1.844	6.321 (3.723-10.996)	4
LDL-C* (mmol/L)			
<=2.98	reference		0
2.99-5.07	0.102	1.107 (0.654-1.852)	1
5.08-8.07	1.576	4.835 (1.477-15.162)	4
BMI** (kg/m^2^)			
<=23.6	reference		0
23.7-29.9	0.777	2.175 (1.255-3.939)	2
30.0-38.0	0.824	2.279 (0.890-5.495)	3
Family history of diabetes			
No	reference		0
Yes	1.250	3.489(1.994-5.979)	4
HDL-C*** (mmol/L)			
>=1.705	reference		0
0.83-1.704	0.956	2.602(1.618-4.268)	3
0.78-0.82	1.381	3.980(0.176-39.921)	4
Diastolic blood pressure (mm Hg)			
<=75	reference		0
76-98	0.297	1.346(0.826-2.244)	2
99-128	1.120	3.064(1.223-7.153)	3
TCHO**** (mmol/L)			
<=5.6	reference		0
>5.6	0.305	1.357(0.737-2.475)	2
Area under the ROC curve	0.811, 95% CI(0.776-0.847)		

* Low Density Lipoproteine Chilostrin

** Body Mass Index

*** High Density Lipoproteine Cholestrin

**** Total Cholestrol

**Figure 1:**
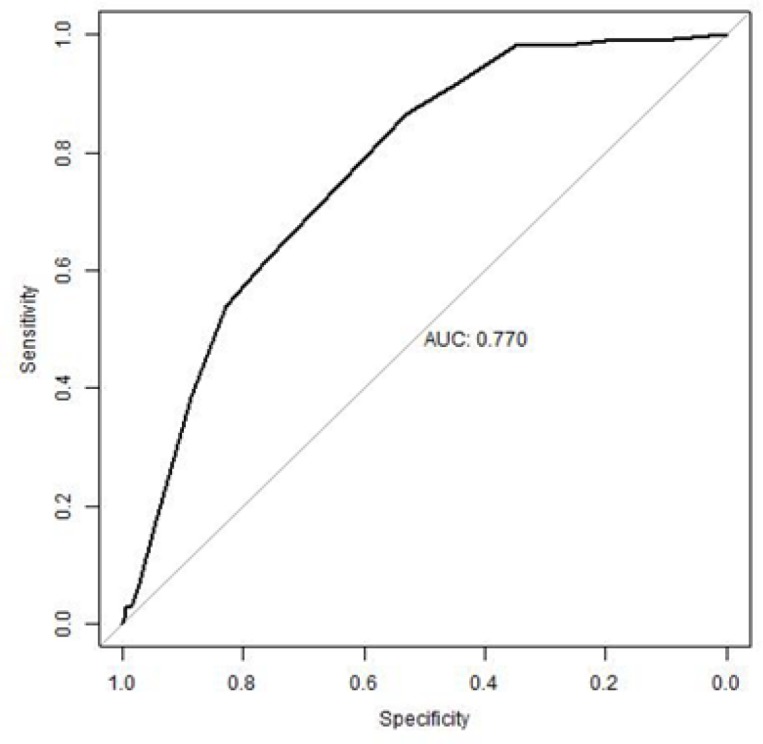
ROC curve of the total risk score for the “Validation population”. The AUC was 0.770 (95%CI: 0.730-0.811).

Finally, we checked the performance of the score model with the “validation population” of 1263 subjects. Among them, 105 (8.31%) subjects were diagnosed with diabetes. We calculated the total score of each subject in the “validation population”, based on the score model developed from the “exploratory population”. The AUC for the total score was 0.770 (95%CI: 0.730-0.811) (fig 1). The AUC’s value was larger than the cutoff value of 0.5 which indicated that the performance of the score model was relatively good for predicting the risk of diabetes for the “validation population”.

## Discussion

In this study, we construted a diabetes score model based on the physcial examination report data. The risk factors we selected for constructing the diabetes score model were age, LDL-C, BMI, family history of diabetes, HDL-C, diastolic blood pressure, TCHO. Based on the calculation of the diabetes score model, we then divided the risk level into four categories: low risk (0-2), middle risk (3-7), high risk (8-12) and extremely high risk (13-17). Validation of the diabetes risk model showed a good performance of the diabetes score model.

Studies have shown that diabetes could have been prevented through the related interventions such as lifestyle intervention or education (25, 26). Therefore, there is a strong favor in screening the potential patients who are at high risk of developing diabetes. Our study is unique that we focused our research on a variety of subject’s demographic and clinical characteristics, which can give a better integrated evaluation of the diabetes risk status. This may provide a simple, practical and useful tool for potential high-risk diabetes individuals to make a proper identification after they received the physical examination reports. The identified high-risk individuals would benefit from receiving health interventions at an early stage so as to prevent the onset of diabetes. It is highly recommended that the high-risk individuals seek appropriate health interventions. Unlike other risk score models developed elsewhere, our research utilized the data from physical examination reports in which the related demographic and clinical data were convenient to be collected from the hospital systems. Compared with other studies, our data collection was easier and it could be applied in our hospitals directly. The score model and its use in self-assessment might be a good way to alleviate the workload of doctors since many of the physical examinations were conducted at the end of the month or year.

HDL-C appeared to be a protective factor in our study. The result was consistent with other studies that HDL-C, a component of the metabolic syndrome, was beneficial to prevent the diabetes. For other risk factors in the score model, a value above the corresponding cutoff value typically indicated a higher risk of diabetes. A major contribution of the integrated score model is that HDL-C was included to capture its preventive function. However, we excluded the drinking and smoking factors in the model development due to possibly oversimplified quantification of these two risk factors. Also, since the information on physical activity and diet was not collected in the physical examination reports, their effects cannot be assessed or taken into consideration into the score model. 

Further research is needed to explore the roles of these factors in risk prediction of diabetes.Compared with the existing diabetes score models (12, 27, 28), our model is innovative in that we stratified the total score into four risk levels, which would make the results easier to be interpreted by the users. More importantly, we tested the performance of the score model through the “validation population”. The validation result confirmed that our risk score model has a good and robust performance in the prediction of the risk of diabetes even though some of the risk factors showed a significant difference between the two groups.

In conclusion, we developed a ready-to-use diabetes risk score model based on the physical examination data which can be applied as a tool to identify individuals at high risk of diabetes. It consisted of the positive predictors, such as age (p<0.0001), LDL-C (p<0.0001), BMI (p=0.0508), family history of diabetes (Yes, p<0.0001), HDL-C (p<0.0001), diastolic blood pressure (p=0.0029), TCHO (p<0.0001), as well as negative predictors TCHO (p<0.0001). People can use it to make a self-assessment based on the data from their physical examination report.
